# Electroacupuncture improves cerebral blood flow and attenuates moderate ischemic injury via Angiotensin II its receptors-mediated mechanism in rats

**DOI:** 10.1186/1472-6882-14-441

**Published:** 2014-11-11

**Authors:** Jing Li, Jiaojun He, Yuanhao Du, Jingjun Cui, Ying Ma, Xuezhu Zhang

**Affiliations:** Department of Acupuncture and Moxibustion Research Institute, First Affiliated Hospital of Tianjin University of Traditional Chinese Medicine, Tianjin, 300193 China; Graduate School, Zhejiang Chinese Medical University, Hangzhou, 310053 China; Department of Neurology, Cangzhou Hospital of Integrated Traditional and Western Medicine of Hebei Province, Cangzhou, 061001 China; Editorial Department, Tianjin University of Traditional Chinese Medicine, Tianjin, 300193 China

## Abstract

**Background:**

To investigate the effects and potential mechanism of electroacupuncture intervention on expressions of Angiotensin II and its receptors-mediated signaling pathway in experimentally induced cerebral ischemia.

**Methods:**

Totally 126 male Wistar rats were randomly divided into control group, model group and EA group. The latter two were further divided into ten subgroups (n = 6) following Middle Cerebral Artery Occlusion (MCAO). Changes in regional cerebral blood flow (rCBF) and expressions of Angiotensin II and its receptors (AT_1_R, AT_2_R), as well as effector proteins in phosphatidyl inositol signal pathway were monitored before and at different times after MCAO.

**Results:**

MCAO-induced decline of ipsilateral rCBF was partially suppressed by electroacupuncture, and contralateral blood flow was also superior to that of model group. Angiotensin II level was remarkably elevated immediately after MCAO, while electroacupuncture group exhibited significantly lower levels at 1 to 3 h and the value was significantly increased thereafter. The enhanced expression of AT_1_R was partially inhibited by electroacupuncture, while increased AT_2_R level was further induced. Electroacupuncture stimulation attenuated and postponed the upregulated-expressions of Gq and CaM these upregulations. ELISA results showed sharply increased expressions of DAG and IP_3_, which were remarkably neutralized by electroacupuncture.

**Conclusions:**

MCAO induced significant increases in expression of Angiotensin II and its receptor-mediated signal pathway. These enhanced expressions were significantly attenuated by electroacupuncture intervention, followed by reduced vasoconstriction and improved blood supply in ischemic region, and ultimately conferred beneficial effects on cerebral ischemia.

## Background

Ischemic stroke is a devastating disease with a complex pathophysiology, and accounts for more than 80% of overall strokes [1]. It often results from focal cerebral ischemia due to occlusion of a cerebral blood vessel, and consequences of blood flow reduction in a brain territory are complex that trigger a serial of multistep pathophysiologic events, the so-called ischemic cascade [2]. The severe reduction of blood flow to the affected tissue results in a lack of oxygen and nutrient transportation, which in turn interferes with intracellular protein synthesis and worsen ischemic brain injury, and ultimately leads to tissue hypoxia and cell death [3]. It is therefore of major important to improve cerebral circulation in acute ischemic stage, and promotion of angiogenesis has been supposed to be a potential therapeutical strategy.

Electroacupuncture (EA) is a novel therapy based on traditional acupuncture combined with modern electrotherapy, and is currently being investigated as a treatment for acute ischemic stroke. Appropriate stimulation of acupoints may increase the blood flow, up-regulate the inherent neuroprotector activity, stabilize the ionic homeostasis, and balance the intracellular survival and death signals in the ischemic brain region. Clinically, EA has been reported to produce beneficial effects on stroke patients, and experimental studies also demonstrated its effective attenuation of cerebral ischemia [4]. Recently, our study has, for the first time, found that EA at GV26 (Shuigou) can not only significantly stimulate endothelial cell proliferation, but also shift its proliferation to an earlier time phase after MCAO, supporting the hypothesis that EA can cause active angiogenesis after MCAO insult and is an important driving force of angiogenesis during cerebral ischemia [5]. However, the underlying mechanism is still an open question and further investigation is required for acute treatment with EA to be widely accepted clinically, as in the present study.

The existence of brain renin-angiotensin system (RAS) has been reported previously, and it has been found to be involved in the modulation of cardiovascular and fluid-electrolyte homeostasis, as well as other brain-specific function. Evidence suggested that RAS blockade may have an impact on early mechanisms of vascular disease, such as endothelial dysfunction and vascular remodeling that underlie clinical manifestations of cardiovascular disease [6]. As a predominate bioactive peptide in RAS, Angiotensin II (AngII) has been suggested to be a significant contributor to the pathophysiology of ischemic stroke [7–9], which, after acting on its receptor (AngII type 1 receptor, AT_1_R; AngII type 2 receptor, AT_2_R ), can activate a series of cell signaling pathways, including phosphatidyl inositol (PI) signaling pathways that associated with vasoconstrictor function of Ang II.

As most understood and physiologically important receptors, AT_1_R has been demonstrated to act through second messengers to promote downstream effects such as vasoconstriction, inflammation, atherogenicity, cellular proliferation and matrix production. Upon binding of the AngII to the AT_1_R, a conformational change is induced and transmitted to the intracellular C-terminus which then interacts with the G protein. Gq, the main G protein associated with AT_1_R, activates phospholipase-C β1 (PLC β1) which in turn activates protein kinase-C causing a release of Ca^2+^ from intracellular stores, increasing cell contractility [10]. In addition to interaction with G proteins, AT_1_R also activates intracellular pathways, such as MAPK and JAK/STAT mechanisms. Ang II binding to AT_1_R causes phosphorylation of PLCγ which cleaves PIP2 to produces IP3 and DAG, followed by Ca^2+^ mobilization and protein kinase C (PKC) activation. These second messengers generated through AT_1_R then contribute to the vasoconstrictor function of Ang II as well as activation of downstream tyrosine and serine/threonine kinases, which contribute to the growth-promoting and cytokine-like actions of Ang II [11, 12]. Long-term blockade of the AT_1_R has been reported to improve the neurological outcome and reduce the infarct volume after experimental focal cerebral ischemia [13].

In contrast, AT_2_R-mediated signaling pathways and function were not very well understood, but in general, appeared to antagonize the effects of the AT_1_R [14]. The AT_2_R has been reported to be highly expressed in fetal tissues, and then restricted to certain locations or only occurs in response to disease [15, 16]. Biochemical and electrophysiological evidence suggested that AT_2_R mediated the angiogenic effect of Ang II through a mechanism that involving bradykinin synthesis, which in turn up-regulates endothelial nitric oxide and prostaglandin, followed by markedly enhanced cerebral blood flow (CBF) and reduced ischemic volume, and ultimately resulted in neuroprotection. It was therefore suggested to represent an important endogenous repair mechanism in promotion of ischemia-induced neovascularization [17, 18].

In view of the significance of Ang II and its receptor proteins in the pathophysiology of ischemic stroke, the present study aimed to explore the potential mechanism mediating the beneficial effects of EA from the respect of Ang II and its receptors-mediated signal transduction pathways, and the time-course effects of EA on experimentally induced cerebral ischemic rats was systematically investigated within the first 24 h.

## Methods

### Study subjects

A total of 126 male Wistar rats (weighing between 180 and 200 g), provided by the Animal Experimental Center of the Medical Sciences Academy of the Chinese People’s Liberation Army Military Academy, were used in the study. All procedures were approved by the Animal Care and Use Committee of Tianjin University of Traditional Chinese Medicine and were in accordance with the institution’s Guidelines for Animal Experiments. Rats were randomly divided into three groups: control group (n = 6), model group (n = 60) and EA group (n = 60). Rats in the latter two groups were further equally subdivided into 10 groups on the basis of difference in time phases, including 1, 2, 3, 6, 9, 12, 15, 18, 21 and 24 h groups. The laser-Doppler examination was only performed on the model and EA groups, with 16 rats in each group.

### Middle cerebral artery occlusion (MCAO) model

The rat MCAO model was established using the intraluminal suture occlusion method as described previously [19]. All animals were fasted overnight with free access to water prior to surgery. During the procedure, room temperature was maintained at 25°C. The animals were subjected to the following set of procedures to induce an infarct. All rats were anesthetized with an intraperitoneal injection of 10% chloral hydrate (0.3 ml/100 g), and an incision was made just in the midline of the neck. The right common carotid artery (CCA), the external carotid artery (ECA) and internal carotid artery (ICA) were carefully isolated, and ligated temporarily at their origin with 6.0 silk suture. Each microaneurysm clip was placed around the CCA and CEA to prevent bleeding during insertion of the suture. A hole of the CCA was then made between the clips with needle (2 ml injector), the blunted tip of a nylon suture (diameter: 0.205 mm; length: 20 ± 2 mm) was inserted through the hole until a mild resistance was felt. The two remaining loose collar sutures were gently tightened, and the vessel clips were then withdrawn. The section was sutured, and rats were administered intravenously with gentamicin (5 mg/kg per day) before put back in cages. Body temperature was maintained between 37 and 37.5°C by means of heating pad. The control group rats (n = 6) were similarly anesthetized and received all the above mentioned surgical procedures except for artery occlusion. All efforts were made to minimize animal suffering.

After MCA occlusion, neurological behaviors were evaluated with Zea Longa’s scale [20]: a score of 0, no neurologic deficit; 1, mild focal neurologic deficit, failure to extend left forepaw fully; 2, moderate focal neurologic deficits, circling to the left; 3, severe focal neurological deficits, falling to the left; 4, did not walk spontaneously and had a depressed level of consciousness; 5, died. Rats that scored 2–3 were deep anesthetized by an intraperitoneal injection of 10% chloral hydrate (0.35 ml/100 g) for further experimentation.

### EA stimulation

The EA stimulation was performed in the EA group as described in our previous study [5]. Briefly, the traditional Chinese acupuncture point ‘Shuigou’ (GV26, No. 26 of the Govern Meridian) was punctured obliquely with 0.5 mm acupuncture needle, and below the GV26 about 1–2 mm, a ground electrode was located and a needle was inserted obliquely in 2 mm depth. All rats in EA groups were stimulated with continued 15 Hz and 1 mA electrical stimulation for 20 minutes.

### Laser doppler detection

Laser Doppler flowmeter (LDF) (DRT4 dual channel, Moor Instruments Ltd, Axminster, UK) was used to monitor regional cerebral blood flow (rCBF) before and at different time points after MCAO. Rats in both model and EA groups were anesthetized with 2% diethyl ether and, after incision of the scalp, a burr hole of 5 mm in diameter was made over the skull at 4 mm posterior coronal suture and 3 mm lateral to the sagittal suture using a low speed dental drill. A laser probe was placed on the dura of cerebral cortex, and the levels of the rCBF were monitored for at least 60 s after blood flow was stabilized. Steady state baseline values were recorded bilaterally before MCAO and all rCBF values were expressed as percentages of respective basal values. Occlusion was confirmed by a drop in LDF by more than 65% of baseline. Before MCAO, the rCBF value was obtained as the baseline, and the rCBF data after MCAO were the percentages of its baseline values.

### Immunohistochemistry assay

In view of the angiogenesis effects of AngII, immunohistochemistry was performed to investigate the level of AngII and its receptor proteins. After the rats were euthanized, ischemic hemisphere tissue was fixed in 10% formalin solution and subsequently embedded in paraffin. A series of 4–6 mm thick sections were cut for up to four sections per animal according to the rat stereotaxic atlas of Paxinos and Watson [21]. Immunohistochemical staining was performed employing a steptavidin- biotin complex (SABC) kit (Wuhan Boster Biological Technology Company, China), using the rabbit polyclonal to Angiopoietin II (1:2000; Abcam Inc., Cambridge, MA, USA), anti-Angiotensin II receptor type1 (1:1000; Sigma, St. Louis, MO, USA), anti-Angiotensin II receptor type2 (1:2000; Sigma, St. Louis, MO, USA), according to the manufacturer's instructions. The microscopic images were captured with a digital camera (Nikon CoolPix 990, Nikon, Tokyo, Japan), and analyzed by Image-pro plus 6.0 software. The ROD was calculated according to the following equation: (OD of Ang II/AT_1_R/AT_2_R − OD of background)/OD of background as reported previously [22, 23].

### Western blotting

All rats fulfilling the inclusion criteria were decollated, and arterial occlusive tissues were snap frozen in liquid nitrogen and stored at −80°C for further use. Western blot analyses were performed to detected concentration of CaM, Gq proteins as described previously with some modifications [24, 25]. Briefly, samples were homogenized in cold lysis buffer, and protein concentrations were determined using a BCA protein assay kit (Beyotime Corporation, China). Cell lysates were analyzed by SDS-PAGE and transferred onto polyvinylidene difluoride (PVDF) membranes. After transfer, membranes were blocked by incubating with 5% (w/v) nonfat dry milk in PBS solution with 0.05% Tween 20 for 1 h at room temperature or overnight at 4°C. Membranes were then incubated with the primary antibodies (anti-calmodulin antibody 1:1000 diluted and anti-GNAQ + GNA11 antibody 1:800 diluted), followed by secondary antibodies (horseradish peroxidase (HRP)-conjugated goat anti-mouse IgG-H&L and HRP-conjugated goat anti-rabbit IgG-H&L), which were all purchased from Abcam (Cambridge, MA, USA), and developed using the enhanced chemiluminescence method (Pierce). Densitometry analysis was carried out using the Quantity One program (Biorad, Hercules, CA, USA).

### Enzyme-linked immunosorbent assay (ELISA)

ELISA was performed to evaluate expressions of the DAG and IP3 as previously described [26]. After the animals were euthanized, brains were perfused with cold saline, and tissue samples of anterior cerebral artery, distributing arteries and origin of posterior cerebral artery were respectively separated and quickly stored at −70°C. Samples were washed in ice-cold PBS and sonicated by a 5-s burst for 5 times with a 90-s cooling period after each burst using an ultrasonic homogenizer (Braun-Sonic 2,000; B. Braun Instruments, Burlingame, Calif.) below 4°C. Samples were then stored at −20°C for coagulation and afterwards incubated in 37°C for 5 min. After the repeated freezing and thawing for 5 times, cell lysates were centrifuged at 1000 g for 20 min, and supernatant was stored at −20°C for ELISA analysis using IP_3_ and DAG ELISA kits (Wuhan EIAab Science Co., Ltd, China).

### Statistical analysis

All data were expressed as the mean ± standard deviation (SD), and statistical analyses were performed with SPSS, version 17.0 (SPSS Inc., Chicago, IL, USA). To establish significance, LDF data was evaluated by two-sample t-test, and the remaining data were analyzed using a one-way analysis of variance (ANOVA). Statistical significance was attributed at a P value of <0.05

## Results

### Neuroethology assessment

Since rats regained consciousness within 3 h postoperatively, neurological assessment was performed from 3 h following the MCAO. As presented in Table 1, neurological behavior scores were decreased with the time in both the model group and EA group. There was no significant difference in the neurological behavior scores at the earlier time points after the surgery (3 and 6 h), while the scores were significantly reduced by EA stimulation at the later time points (9, 15, 18, and 21 h after MCAO insult), when compared with the model group at the same time points (*p* < 0.05).

**Table 1:** **Neurological behaviors were evaluated with Zea Longa’s scale after MCA occlusion**

Time/h	Model group	EA group
3	2.83 ± 0.47	2.83 ± 0.47
6	2.83 ± 0.41	2.67 ± 0.52
9	2.67 ± 0.52	2.50 ± 0.84^※^
12	2.50 ± 0.55	2.33 ± 0.52
15	2.33 ± 0.82	2.00 ± 1.1^※^
18	2.17 ± 0.98	2.00 ± 0.89^※^
21	2.17 ± 0.75	1.83 ± 0.89^※^
24	2.17 ± 0.75	1.83 ± 0.75

MCA: Middle cerebral artery; ^※^ *p* < 0.05 vs the model group at the same time points.

### Laser doppler analysis of rCBF level

LDF was used to determine the rCBF levels in the both cerebral infarction ipsilateral and contralateral to the lesion site, results showed that rCBF values were reduced to various degrees after MCAO insult (Table 2).

**Table 2:** **Time-course effects of EA intervention on rCBF levels in both the cerebral infarction ipsilateral and contralateral to the lesion site**

Time h	Model group		EA group	
	Ipsilateral tissue	Contralateral tissue	Ipsilateral tissue	Contralateral tissue
Pre-operation	1.00	1.00	1.00	1.00
0	0.30 ± 0.17	0.76 ± 0.30	0.29 ± 0.16	0.80 ± 0.17
1	0.34 ± 0.13	0.85 ± 0.33	0.49 ± 0.15^※^	0.83 ± 0.20
2	0.35 ± 0.11	0.93 ± 0.23	0.52 ± 0.25^※^	0.82 ± 0.33
3	0.47 ± 0.16	1.07 ± 0.41	0.61 ± 0.34^※^	0.96 ± 0.39
6	0.70 ± 0.23	1.15 ± 0.49	0.67 ± 0.19	1.31 ± 0.62
9	0.70 ± 0.25	1.01 ± 0.33	0.69 ± 0.32	1.22 ± 0.40
12	0.68 ± 0.32	1.15 ± 0.33	0.82 ± 0.25^※^	1.20 ± 0.47
15	0.73 ± 0.30	0.95 ± 0.28	0.66 ± 0.25	1.15 ± 0.49
18	0.75 ± 0.27	0.91 ± 0.52	0.75 ± 0.22	1.08 ± 0.51
21	0.58 ± 0.20	0.98 ± 0.28	0.68 ± 0.14	1.24 ± 0.41^※^
24	0.65 ± 0.31	0.69 ± 0.36	0.61 ± 0.27	1.08 ± 0.74^※^

rCBF: regional cerebral blood flow; EA: electroacupuncture; ^※^ *p* < 0.05 vs the corresponding model group at the same time points.

In the cerebral infarction ipsilateral tissue, a remarkable decline in the rCBF level was observed immediately after MCAO (0.30 ± 0.17of the baseline), and level was then slightly increased 1 to 6 h after MCAO (Table 2). During the 18 to 24 h time phase, value was declined to some extent, but still higher than the baseline. The same tendency existed in EA group, where rCBF value was declined to 0.29 ± 0.16 of the baseline. However, at the time interval from 1 to 6 h after MCAO, EA treatment resulted in a transient and significant suppression of the MCAO-induced decline in rCBF levels (*p* < 0.05) As shown in Table 2, EA treatment transiently raised the decreased rCBF to (0.49 ± 0.15) of the baseline at 1 h after MCAO, and value was then gradually increased until 6 h time phase.

As for contralateral side, rCBF value was only reduced by 0.22 ± 0.24 immediately after MCAO in both the model and EA groups, and was slightly increased at the time intervals from 1 to 6 h. However, after the 6 h time phase, CBF perfusion in EA group was found to be significantly increased and became higher than that of control and model groups. Although there was no statistical significance in the changes of rCBF levels during the different time phases, the levels were generally higher in EA group than that of model group, especially at the time interval from 21 and 24 h (*p <* 0.05, Table 2).

### Immunohistochemistry assay

**Figure 1:**
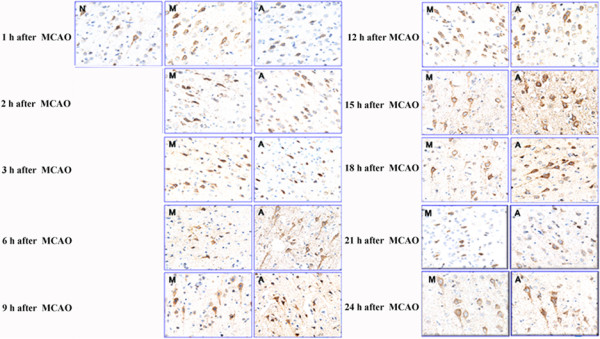
**Time-course effects of EA intervention on the expression of Ang II was investigated by immunohistochemistry assay in brain tissue after MCAO.** Positive expressions of Ang II and its receptors were generally identified in the brain tissue. After MCAO insult, a remarkable augmentation in Ang II level was observed immediately after MCAO, and reached a peak at 12 h following MCAO (*p* < 0.05). The same tendency was obtained in EA group. Which, however, exhibited a significantly lower level at the 1 to 3 h, and after that, the value was significantly increased and peaked at 24 h. N: Control group; M: Model group; A: EA group.

As presented in Figure 1 and Table 3, a remarkable augmentation in AngII levels was observed immediately after MCAO (*p* < 0.05), and reached a peak at 12 h after MCAO insult. At the time phase from 21 to 24 h, the level tended to grow again. The same tendency was obtained in EA group. Which, however, exhibited a significantly lower level at 1 to 3 h time phase when compared with model group, and the value was significantly increased until 15 h and then slightly reduced thereafter (*p* < 0.05). However, it exhibited a rising trend again at 21 to 24 h after MCAO, with the maximal value obtained at 24 h time phase.

**Table 3:** **Time-course effects of EA intervention on the expressions of Ang II, AT**_**1**_ **R, and AT**_**2**_ **R after MCAO as determined by immunohistochemistry assay (IOD)**

Time h	Model group			EA group		
	AngII	AT _1_R	AT _2_R	AngII	AT _1_R	AT _2_R
1	2.139 ± 0.295^※^	2.768 ± 0.454^※^	1.912 ± 0.484	1.746 ± 0.336^**^	2.474 ± 0.428^※*^	2.373 ± 0.487^**^
2	2.200 ± 0.177^※^	2.777 ± 0.222^※^	1.952 ± 0.505	1.928 ± 0.268^**^	2.317 ± 0.409^※**^	2.501 ± 0.449^※**^
3	2.259 ± 0.215^※^	2.496 ± 0.145^※^	2.455 ± 0.297^※^	2.003 ± 0.287^**^	2.191 ± 0.553^※*^	2.780 ± 0.339^※**^
6	2.330 ± 0.528^※^	2.425 ± 0.396^※^	2.526 ± 0.632^※^	2.634 ± 0.474^※**^	1.956 ± 0.366^※**^	2.885 ± 0.583^※*^
9	2.667 ± 0.383^※^	2.379 ± 0.496^※^	2.599 ± 0.454^※^	2.901 ± 0.344^※*^	2.057 ± 0.438^※*^	3.117 ± 0.412^※**^
12	2.889 ± 0.257^※^	2.595 ± 0.281^※^	2.484 ± 0.533^※^	3.108 ± 0.193^※*^	2.351 ± 0.193^※**^	3.155 ± 0.443^※**^
15	2.773 ± 0.344^※^	2.604 ± 0.262^※^	2.661 ± 0.428^※^	3.226 ± 0.210^※*^	2.337 ± 0.475^※*^	3.346 ± 0.295^※**^
18	2.754 ± 0.245^※^	2.657 ± 0.280^※^	2.539 ± 0.618^※^	3.012 ± 0.308^※**^	2.448 ± 0.366^※*^	2.997 ± 0.594^※*^
21	2.376 ± 0.542^※^	2.744 ± 0.436^※^	2.400 ± 0.623^※^	2.669 ± 0.479^※*^	2.526 ± 0.257^※*^	2.882 ± 0.588^※**^
24	2.474 ± 0.463^※^	2.697 ± 0.321^※^	2.445 ± 0.494^※^	3.233 ± 0.335^※**^	2.365 ± 0.400^※**^	2.817 ± 0.513^※*^

EA: Electroacupuncture; Ang II: Angiotensin II; AT_1_R: Ang II type 1 receptor; AT_2_R: Ang II type 2 receptor; ^※^ *p* < 0.05 vs the control group (values were 1.872 ± 0.331 for AngII, 2.037 ± 0.307 for AT_1_R, and 2.091 ± 0.451 for AT_2_R), ^*^ *p* < 0.05, ^**^ *p* < 0.01 vs model group at the same time points.

**Figure 2:**
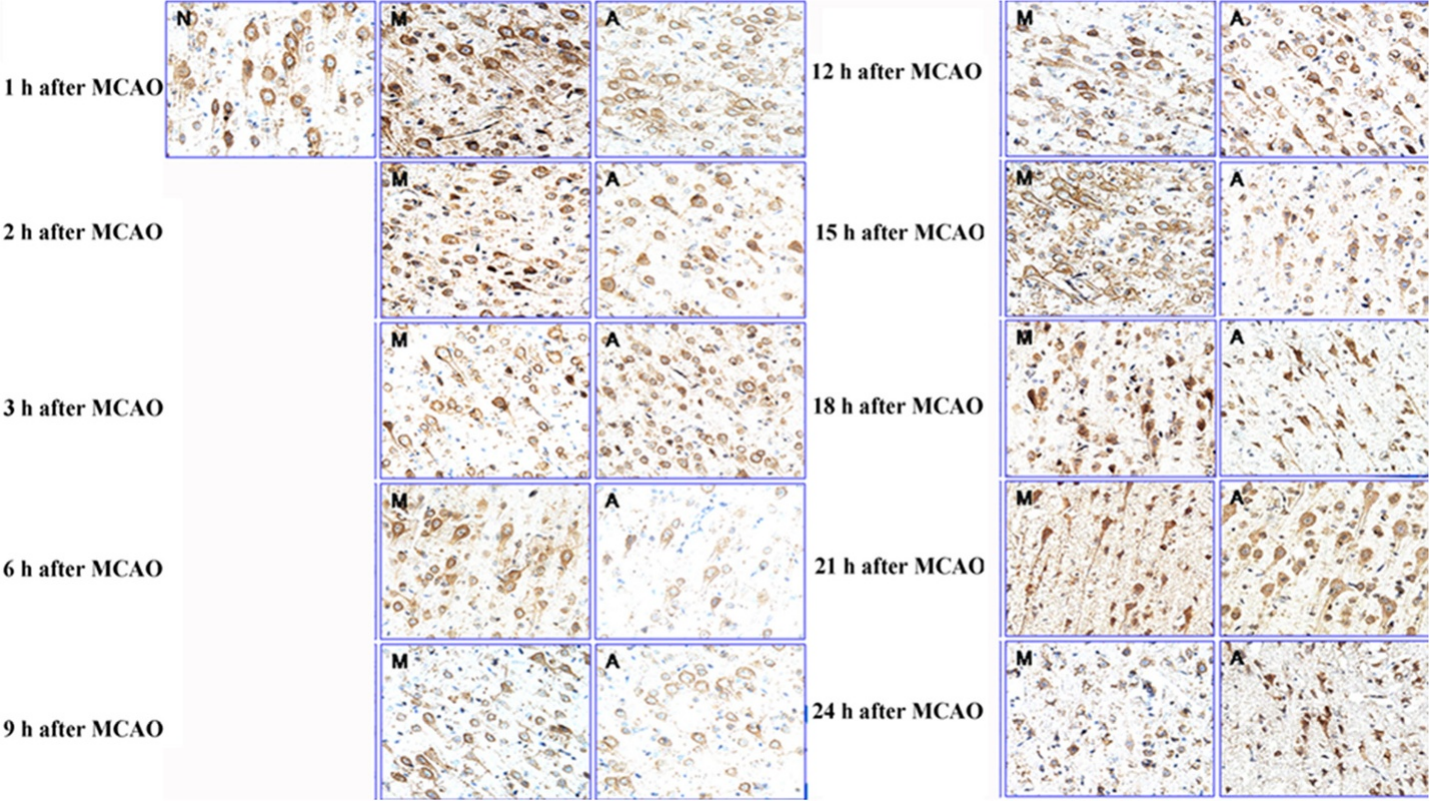
**Time-course effects of EA intervention on the expression of AT1R was investigated by immunohistochemistry assay in brain tissue after MCAO.** MCAO induced significant increase in protein level of AT1R, and the effect was partially inhibited by EA treatment. N: Control group; M: Model group; A: EA group.

Changes in expression of AT_1_R and AT_2_R were also analyzed immunohistochemically. Results showed that MCAO induced a remarkable up-regulation of AT_1_R level in model group (*p* < 0.05), and the value was remarkably reduced by EA treatment at all time phases after MCAO, when compared with the model group counterparts (*p* < 0.05; Figure 2 and Table 3). The maximal value of AT_1_R was obtained at 2 h following MCAO, and then gradually decreased until 12 h. However, the value tended to increase again after the 15 h time phase.

**Figure 3:**
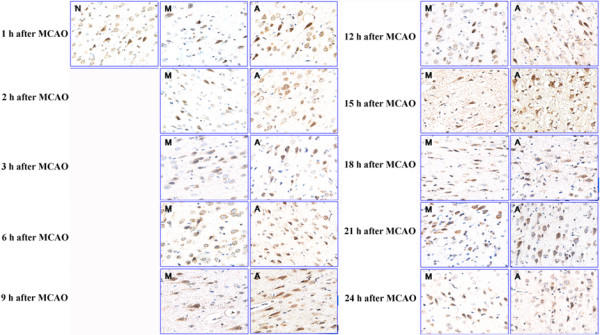
**Time-course effects of EA intervention on the expression of AT2R was investigated by immunohistochemistry assay in brain tissue after MCAO.** MCAO induced transient decrease in protein level of AT_2_R, while AT_2_R expression was found to be significantly elevated after EA stimulation. N: Control group; M: Model group; A: EA group.

Figure 3 and Table 3 showed changes in AT_2_R expression at different time phases after MCAO. Expression of AT_2_R was reduced to some extent during the time interval from 1 to 2 h, and then increased sharply thereafter (*p* < 0.05). In EA group, however, AT_2_R level was found to be always higher than that of control and model groups (*p* < 0.05).

### Western blotting analysis of CaM and Gq expressions

**Figure 4:**
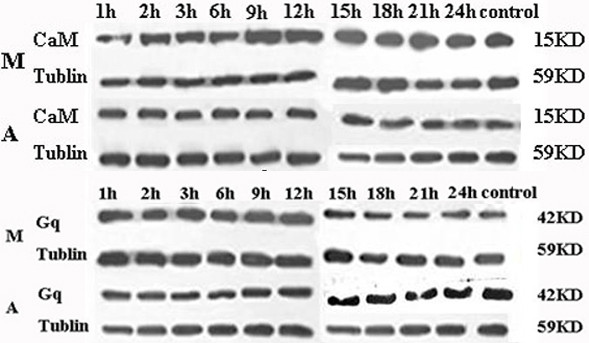
**Time-course effects of EA intervention on the CaM and Gq expressions.** Western blotting results showed that MCAO induced significant increases in CaM levels, and this effect was significantly suppressed by EA intervention. While, MCAO insult induced a transient increase in expression of Gq, and EA stimulation not only attenuated these MCAO-induced up-regulations to varying degrees, but also shifted these upregulations to the later time phases.

Western Blotting assay showed that CaM level was significantly higher at all the time phases after MCAO (Figure 4). EA treatment induced a significant decline in CaM level at 1, 2, 3, 6, 9, 12, 15 and 21 h time phase following MCAO when compared with the model group, although it was still higher than the control (*p* < 0.05; Table 4).

**Table 4:** **Time-course effects of EA intervention on the expressions of CaM and Gq as determined by western blot assay**

Time h	Model group		EA group	
	CaM	Gq	CaM	Gq
1	0.67 ± 0.03^※^	0.86 ± 0.05^※※^	0.58 ± 0.06^**^	0.49 ± 0.27^**^
2	0.74 ± 0.09^※※^	1.33 ± 0.40^※※^	0.59 ± 0.07^**^	0.53 ± 0.17^**^
3	0.78 ± 0.21^※※^	1.50 ± 0.41^※※^	0.63 ± 0.12^**^	0.90 ± 0.12^※※**^
6	0.82 ± 0.16^※※^	1.42 ± 0.34^※※^	0.70 ± 0.10^※*^	1.02 ± 0.30^※※**^
9	0.88 ± 0.20^※※^	0.96 ± 0.22^※※^	0.72 ± 0.17^※**^	0.65 ± 0.21^**^
12	0.95 ± 0.08^※※^	0.64 ± 0.06	0.75 ± 0.12^※**^	0.64 ± 0.36
15	0.94 ± 0.09^※※^	0.59 ± 0.15	0.76 ± 0.15^※※**^	0.63 ± 0.08
18	0.89 ± 0.05^※※^	0.53 ± 0.18	0.82 ± 0.18^※※^	0.53 ± 0.12
21	0.92 ± 0.09^※※^	0.50 ± 0.09	0.78 ± 0.19^※※*^	0.42 ± 0.07
24	0.88 ± 0.05^※※^	0.58 ± 0.29	0.81 ± 0.11^※※^	0.53 ± 0.12

EA: Electroacupuncture; Gq: Gq protein; CAM: Calcium/calmodulin-dependent protein; ^※^ *p* < 0.05, ^※※^ *p* < 0.01 vs control group (values were 0.55 ± 0.07 for CaM and 0.44 ± 0.10 for Gq), ^*^ *p* < 0.05, ^**^ *p* < 0.01 vs model group at the same time points.

Expression of Gq was also increased after MCAO when compared with the control group, however, the tendency was quite different from that of the CAM. A robust elevation in Gq protein levels was observed 1–3 h following MCAO insult (*p* < 0.01; Table 4). The value was significantly higher in model group than that in EA group at the 1, 2, 3, 6, and 9 h time phase, and after that, there was no statistical difference in the Gq expression. Although Gq level was elevated in EA group when compared with the control group, it was delayed and blunted when compared with the model group. Gq level of the 1 and 2 h after MCAO was consistent with that of control group (*p* > 0.05), and only during the 3 and 6 h time phases it was increased to some extent and then decreased thereafter.

### ELISA assay of expressions of DAG and IP3

Expression of DAG and IP3 was analyzed by using ELISA assay, results showed that during the 1 to 12 h time interval, MCAO induced a sharp increase in DAG level in model group, with the peak value reached at 12 h after MCAO (*p* < 0.05; Table 5). While EA treatment partially neutralized this increase, only the 9 h DAG level was significantly higher than that of the control group (*p* < 0.05), and after that, the value was declined and during the 15 to 24 h time phase, it was significantly lower than that of control and model groups (*p* < 0.05).

**Table 5:** **Time-course effects of EA intervention on the expressions of DAG and IP3 as determined by ELISA assay**

Time h	Model group		EA group	
	DAG(ng/mL)	IP _3_(ng/mL)	DAG(ng/mL)	IP _3_(ng/mL)
1	1.903 ± 0.122^※^	2.045 ± 0.097^※^	0.956 ± 0.192^**^	0.857 ± 0.117^**^
2	2.160 ± 0.274^※^	2.144 ± 0.096^※^	0.787 ± 0.185^**^	1.078 ± 0.061^**^
3	2.304 ± 0.177^※^	2.420 ± 0.094^※^	1.524 ± 0.24^※**^	1.082 ± 0.079^※**^
6	2.480 ± 0.194^※^	2.489 ± 0.131^※^	1.500 ± 0.13^※**^	1.162 ± 0.094^※**^
9	3.096 ± 0.186^※^	2.760 ± 0.151^※^	1.332 ± 0.463^※*^	1.203 ± 0.196^※**^
12	3.662 ± 0.157^※^	3.309 ± 0.126^※^	1.741 ± 0.188^※*^	1.317 ± 0.093^※**^
15	1.017 ± 0.128	2.387 ± 0.123^※^	0.614 ± 0.04^※**^	1.185 ± 0.100^※**^
18	1.110 ± 0.136	2.244 ± 0.052^※^	0.562 ± 0.08^※**^	0.855 ± 0.09^※**^
21	0.924 ± 0.047	1.801 ± 0.040^※^	0.506 ± 0.085^※*^	0.708 ± 0.139^*^
24	0.813 ± 0.029	1.784 ± 0.162^※^	0.276 ± 0.12^※**^	0.553 ± 0.035^**^

EA: Electroacupuncture; ELISA: Enzyme-linked immuno sorbent assay: DAG: Diacylglycerol; IP_3_: Inositol 1, 4, 5-trisphosphate; ^※^ *p* < 0.05 vs control group (values were 0.982 ± 0.134 for DAG and 0.721 ± 0.081 for IP_3_), ^*^ *p* < 0.05, ^**^ *p* < 0.01 vs model group at the same time points.

In model group, expression level of IP_3_ was also significantly increased immediately after MCAO (*p* < 0.05), and reached the peak value at 12 h, coincident in time with that of DAG expression (Table 5). The same tendency existed in EA group, where, however, the IP_3_ value was significantly reduced by EA treatment at all time phases when compared with model group counterparts (*p* < 0.05).

## Discussion

Animal modeling of ischemic stroke serves as an indispensable tool first to investigate mechanisms of ischemic cerebral injury. As an ideal subject for mimicking human stroke, rat has been largely used in molecular pathophysiology of stroke. However, some studies have demonstrated that certain rat strains are more suitable to use in stroke models, for instance low deformity rates at the cerebrovascular differentiation make Wistar rats more suitable for suture MCAO than SD rats, while Wistar rats weighing between 180 and 200 g showed comparatively low deformity rates. Besides, female rats have been suggested to sustain smaller infarcts after MCAO compared with male rats and this gender difference may be affected by female hormone [27]. Put these all together, male Wistar rats weighing between 180 and 200 g were selected in the present study.

The extent of postischemic injury depends not only on the degree and duration of arterial occlusion, but also on the adequacy of collateral circulation. After cerebral infarction, improvement in collateral circulation generally includes two aspects, early vasodilation (initial of collateral circulation) and subsequent angiogenesis (collateral circulation reconstruction). Our recent study showed that EA stimulation can significantly increase the endothelial cell proliferation at the acute stage of the cerebral infarction (first 6 h after MCAO), when compared with model group. Meanwhile, the initial proliferation was reported to shift from 24 h in model group to an earlier time of 12 h in EA group. With that, we believed that vasodilation- dependent improvement in collateral circulation alone may sustain to 24 h after MCAO, and hence, the present study was systematically performed at 1, 2, 3, 6, 9, 12, 15, 18, 21 and 24 h time phases after MCAO insult.

EA treatment could induce protective effects on ischemic patients, but there are some characters defining the protective effect of EA treatment, especially the electrical stimulation parameters and the acupoint specificity. Acupoint selection is the determining factor of EA treatment, different acupoints has been employed previously to investigate the acupoint-specific beneficial effects [28]. Based on meridian theory, an acupoint is relatively specific to certain functions or certain organs, and different effects occur when different acupoints are stimulated. As the traditional Chinese acupuncture point, GV26 was specific for enhancement of CCA blood flow [14], and stimulation at GV26 supposed to improve the functions of CBF vessel via nerve reflex pathway [29].

Previous studies showed evidence suggested that electric stimulation of different parameters may have different effects on some body functions [30]. According to the nested design study on EA combined with our earlier research [30], stimulation parameters, with a stimulation rate of 15 Hz and an electrical current amplitude of 1 mA for 20 minutes, was adopted in this study.

Stenosis and occlusion of the MCA was supposed to be the most important factors responsible for intracranial ischemic lesions, and hemodynamic lesions in acute cerebral infarction area were primarily characterized by decline in the CBF and cerebral blood volume (CBV) [31]. Cerebral blood vessel has been reported to exhibit self-regulation effects, when the CBF perfusion pressure was fluctuated within a certain range, CBF level was maintained by the compensatory expansion or contraction of the small artery and blood capillary smooth muscle. However, when the collateral circulation and small blood vessels expansion were above a certain limit, CBF level began to fall and further developed into malignant perfusion lesions despite of normal or slightly elevated CBV. Where cerebrovascular reserve capacity was evidently disturbed on the occluded side, followed by the significantly reduced CBF and CBV values, and ultimately developed into stroke [32]. Previous researches showed that rats with the rCBF reduction to less than 45% of the threshold level had a higher possibility (80%) of development into the medium-sized infarcts, while reduction less than 55% would inevitably resulted in the small-sized infarcts [33]. Cerebral infarction posed serious obstacle to cerebrovascular function and morphology, and stimulation at GV26 was suggested to promote diastolic blood vessel, reduce the cerebrovascular spasm, improve cerebrovascular self-discipline and energy metabolism, and thereby promote the contralateral CBF to the infarction side to provide timely and effectively compensate for blood flow.

Based on the above mentioned principle, the effects of EA intervention on Ang II and its receptor-mediated signal pathway were systematically evaluated in this study. LDF analysis of the rCBF showed that MCAO induced a significant decline in rCBF level of the infarct hemisphere when compared with the contralateral side (*p* < 0.05), with the rCBF level reduced to the (0.30 ± 0.17) and (0.29 ± 0.16) of the baseline, respectively. While EA treatment resulted in a transient and significant up-regulation of rCBF level at the acute stage of cerebral infarction, especially at 1 h (0.49 ± 0.15), when compared with model group (*p* < 0.05). After that, there was no significant difference in rCBF levels between model and EA groups, although they were still lower than that of the control group (*p* < 0.05). Our results indicated that stimulation at GV26 can rapidly increase rCBF level to up to 45% of the threshold, and therefore improve rCBF perfusion promptly and efficiently, resulting in the reduction or totally avoid of ischemia or infarction in the affected area.

Activation of the RAS has been linked with an increased risk of myocardial infarction and stroke [34, 35], and increasing amount of data showed that Ang II may be involved in the initiation and regulation of processes occurring in brain ischemia either in animal models of cerebral ischemia [36] or in stroke patient [37]. The vast majority of physiological and pathophysiological effects of Ang II occurred via the AT_1_R, and they were widely distributed in the brain tissue. In contrast, the AT_2_R is highly and ubiquitously expressed in certain cerebral tissue, and its re-expression only occurs in response to disease [15]. In the present study, the positive expression of AT_2_R has been found in ischemic core and penumbra regions, corroborating the results reported by Makino I [38].

Previous studies on the expression of AngII and its receptors mainly focused on the 24, 48 and 72 h time phases after MCAO [39], and systemic evaluation of their expression within the first 24 h is scarce. Therefore, in this study, a continuous and dynamic evaluation of changes in the first 24 h following MCAO was established. The result showed that at the time intervals from 1 to 24 h after MCAO, AngII level was significantly higher than that of control. The values was significantly higher in EA group after 6 h time phase when compared with model group, and peak values were reached at 12 and 24 h in model and EA group, respectively. It may suggest that ischemia and hypoxia can stimulate the up-regulation of AngII, but this self-regulation is relatively limited without external intervention.

In our opinion, this phenomenon revealed that animal organism has an ability of self-restoration in hypoxic–ischemic state, but this ability only has limited benefit to cerebral ischemia as described in our experiment. With the development of ischemic process, disturbed protein synthesis and cell degeneration or necrosis would inevitably result in the above-mentioned phenomenon.

The previous results on AT_1_R expression were controversial [9, 40, 41], although the underlying reason was still unknown, it may be because of different observation time and places. After the systematic evaluation of AT_1_R level at the cerebral cortex (both brain parenchyma and blood vessels) during the first 24 h after MCAO, present study showed that AT_1_R level was significantly higher in the model group, and peaked at 2 h time phase. The value was declined to some extent during the 3 to 12 h, and then tended to increase again. It may be resulted from the self-regulation and stimulation effects of the AngIIon AT_1_R expression. Previous studies showed that expression of the AT_2_R mRNA level was up-regulated at the 3 and 24 h after cerebral infarction. However, a remarkable upregulation of the AT_2_R level, starting at 3 h and peaking at 15 h after MCAO insult, was observed in the present study (*p* < 0.05).

Early cerebral infarction is generally characterized by vasospasm, with less blood vessels on the brain surface. AT_1_R was widely present in the brain, and was suggested to mediate the vasoconstriction and endothelial cell injury effects of AngIIthrough stimulation of the IP_3_ signal transduction pathway [4]. Recently identified effects of the AT_1_R include production and release of reactive oxygen species, stimulation of inflammatory and thrombotic processes, which contribute to Ang II–mediated inflammation and atherogenesis [42, 43]. Our findings presented here indicated that EA at GV26 can down-regulated the expression of AT_1_R, which in turn promote vasodilatation, followed by improved CBF and ultimately resulted in the neuroprotective effect of EA. On the other hand, the results revealed that AT_1_R levels increased during 1 to 6 h following MCAO, and minimum expression was at 6 h, suggesting that beneficial effects of EA treatment may exhibit a time response. The findings of our study may therefore provide certain scientific basis for the future clinical treatment.

Antagonism to the function AT_1_R, AT_2_R was supposed to mediated the angiogenic effect of Ang II through a mechanism that involves bradykinin [17, 18], followed by up-regulation of endothelial NO and prostaglandin, which in turn markedly enhanced CBF and reduced ischemic volume, and ultimately resulted in neuroprotection after MCAO. The data in this study showed that up-regulation of AT_2_R expression was presented in both model and EA treated animals. However, the value was significantly higher in EA group, and this up-regulation was also observed to start earlier (2 h after MCAO) when compared with model group (3 h after MCAO), indicating that stimulation at GV26 can not only up-regulate expression of AT_2_R, but also shift its up-regulation to an earlier time.

Put these all together we can see that AngIIexhibited a vasoconstriction effect via AT_1_R, the primary characteristic of early cerebral infarction. EA treatment at GV26 inhibited AngIIexpression, which in turn reduced its combination with AT_1_R and thereby alleviated vasoconstriction. With the development of ischemia, the damage factors such as peroxide and oxygen free radicals were accumulated in the brain, along with the enhanced expression of AT_2_R, which in turn promoted its combination with the upregulated AngII, and ultimately improved the cerebral ischemia.

However, this regulation effect only had the limited benefit, and EA treatment can not reverse cerebral ischemia. With the development of the ischemia and increase in cells degeneration or necrosis, AngIIlevel would inevitably decline. Whereas the value tended to increase again at the 24 h time phase, it may be because that EA treatment can promote the growth of glial cells, which not only replaced the necrotic cells, but also acted as the primary synthetic cells that stimulating AngIIexpression. Our results suggested that stimulation at GV26 can regulate the expression of AngIIto exert beneficial effects on cerebral infarction.

AngII, when bound to AT_1_R, would initiate mechanisms that would increase heart contractile force, vascular tone, and constriction of vessels [7]. AT_1_R was suggested to act through second messengers to promote downstream effects such as vasoconstriction, atherogenicity, cellular proliferation and matrix production, while AT_2_R expression was supposed to be restricted to certain locations or only occurred in response to disease [15, 16].

Therefore, in addition to evaluation of changes in AngIIand its receptors levels, expression of the effector proteins in the AngIIreceptor mediated IP_3_ signal transduction pathway were also determined in this study. Western blotting results showed that MCAO induced a significant increase in Gq and CaM expressions, although different tendencies could been seen between the two values. Briefly, CaM level was significantly higher at all time phases after MCAO, and EA treatment induced a pronounced suppression of CaM expression, although it was still higher than control (*p* < 0.05). By contrast, a robust elevation in Gq level was observed mainly at 1 to 3 h following MCAO insult, EA treatment not only attenuated the MCAO-induced enhancement of Gq expression, but also shifted it to a later time at 6 h after MCAO (*p* < 0.05). ELISA assay was performed to determine the amounts of DAG and IP3 produced after MCAO, results showed that expression levels of DAG and IP3 were sharply increase 1–12 h following MCAO, and peak expression was at 12 h (*p* < 0.05). After EA treatment, however, these increases were remarkably neutralized when compared with model group. The results presented here suggested that MCAO induced a sharp increase in expression of the effector proteins in AngII receptor mediated IP signal transduction pathway. These enhanced expressions were significantly suppressed by EA treatment, followed by the reduction of the MCAO-induced vasoconstriction, which in turn improved blood supply in the ischemic region, and ultimately exerted beneficial effects on cerebral ischemia.

## Conclusions

Stimulation at GV26 may prove to be useful in partially reverse the MCAO-induced up-regulations of AngII and its receptor mediated IP3 signal transduction pathway, followed by reduced vasoconstriction and improved blood supply in ischemic region, and ultimately conferred beneficial effects on cerebral ischemia.
